# Genome sequence data of *Bacillus velezensis* BP1.2A and BT2.4

**DOI:** 10.1016/j.dib.2022.107978

**Published:** 2022-02-22

**Authors:** Christian Blumenscheit, Jennifer Jähne, Andy Schneider, Jochen Blom, Thomas Schweder, Peter Lasch, Rainer Borriss

**Affiliations:** a Proteomics and Spectroscopy Unit (ZBS6), Center for Biological Threats and Special Pathogens, Robert Koch Institute, Berlin, Germany; b Bioinformatics and Systems Biology, Justus-Liebig Universität Giessen, Giessen, Germany; c Institute of Marine Biotechnology e.V. (IMaB), Greifswald, Germany; d Nordreet UG Greifswald, Germany

**Keywords:** Complete genome, Phylogenetic analysis, *Bacillus velezensis*, Lipopeptides, Polyketides, Macrolactin

## Abstract

Here, we report the complete genome sequence data of the biocontrol strains *Bacillus velezensis* BP1.2A and BT2.4 isolated from Vietnamese crop plants. The size of the genomes is 3,916,868 bp (BP1.2A), and 3,922,686 bp (BT2.4), respectively. The BioProjects have been deposited at NCBI GenBank. The GenBank accession numbers for the *B. velezensis* strains are PRJNA634914 (BP1.2A) and PRJNA634832 (BT2.4) for the BioProjects, CP085504 (BP1.2A) and CP085505 (BT2.4) for the chromosomes, GCA_013284785.2 (BP2.1A), and GCA_013284785.2 (BT2.4) for GenBank assembly accessions, and SAMN15012571 (BP1.2A) and SAMN15009897 (BT2.4) for the BioSamples. Both genomes were closely related to FZB42, the model strain for plant growth promoting bacilli.

## Specifications Table

**Table:** 

Subject	Biological sciences
Specific subject area	Molecular Phylogenetics
Type of data	Table, Figure, genome sequencing data in FASTA format.
How the data were acquired	Short reads were generated with Illumina HiSeq at LGC Genomics (Berlin, Germany). Long reads were obtained with Oxford Nanopore MinION.
Data format	Analyzed DNA sequence data in FASTA, NEWICK and text format.
Description of data collection	Pure cultures of BP1.2A and BT2.4 were used to isolate genomic DNA and to obtain the genomic data. Genome annotation was carried out using NCBI Genome Automatic Annotation Pipeline (PGAP) and RAST.
Data source location	BP1.2A was isolated from black pepper roots (Viet Nam; Chu Se, Gia Lei), and BT2.4 was isolated from dragon fruit tree (Viet Nam: Ham thuan Nam, Binh Thuan) by • Le Thi Thanh Tam, PPRI • Hanoi • Viet Nam
Data accessibility	The BioProjects have been deposited at NCBI GenBank under the following accession numbers: Bioprojects: PRJNA634914 (BP1.2A), and PRJNA634832 (BT2.4), Biosamples: SAMN15012571 (BP1.2A), and SAMN15009897 (BT2.4), Sequences of the chromosomes: CP085504.1 (BP1.2A) and CP085505.1 (BT2.4), GenBank assembly accessions: GCA_013285085.2 (BP1.2A), and GCA_013284785.2 (BT2.4). The SRA records could be accessed for BP1.2A, and BT2.4 from their corresponding links from the BioProjects. https://www.ncbi.nlm.nih.gov/sra/PRJNA634914 https://www.ncbi.nlm.nih.gov/sra/PRJNA634832
	With the article
	L.T.T. Tam, J. Jähne, P.T. Luong, L.T.P. Thao, L.T.K. Chung, A. Schneider, C. Blumenscheit, P. Lasch, T. Schweder, R. Borriss. Draft genome sequences of 59 endospore-forming Gram-positive bacteria associated with crop plants grown in Vietnam. Microbiol. Resour. Announc. 9 (2020): e01154–20 https://doi/10.1128/MRA.01154–20.

## Value of the Data

• The data of this article demonstrate that it is possible, to isolate closely related *Bacillus* strains from remote geographical regions with different climatic conditions
• BP1.2A, and BT2.4 share 99.99% identical residues with the model strain FZB42 (Table 3). The high similarity of the two novel strains with the biocontrol strain FZB42, encourages the development of the strains as promising biocontrol agents used in sustainable agriculture in temperate and subtropical zones, as well.
• The data demonstrate that gene clusters involved in non-ribosomal and ribosomal synthesis of antibacterial and antifungal secondary metabolites are highly conserved in different representatives of *B. velezensis*, despite of their geographical distribution.
• For the scientific community, the genome data presented here, extend the resources for comparative genomic analysis among the members of the *Bacillus amyloliquefaciens* operational group, including *Bacillus velezensis*, at present the most important species used in biological plant protection.
• Furthermore, extended genomic analyses performed between closely related bacteria should elucidate regions and/or genes with different variability and might identify regions (genes) with an enhanced mutation bias.

## 1 Data Description

The draft genome sequences of 59 Gram-positive bacterial strains that were isolated from Vietnamese crop plants have been already reported [1]. Two of these strains, *B. velezensis* BP1.2A, and *B. velezensis* BT2.4, were now completely sequenced using the nanopore sequencing technology. Both sequences exhibited a very high degree of similarity with the model strain of plant-growth promoting Gram-positive bacteria, *B. velezensis* FZB 42 [2].

The complete genomes consist of single circular chromosomes with 3916,868 bps (BP1.2A) and 3922,686 bps (BT2.4), respectively. Automatic genome annotation was performed using the RAST (Rapid Annotation using Subsystems Technology) server [3], and the NCBI Genome Automatic Annotation Pipeline (PGAP) [4] for the general genome annotation deposited in NCBI.

As shown in Table 1, subsystem proteins distribution [5] of the two strains is very similar to FZB42 [6] indicating their close relationship. Genome mining of *B. velezensis* performed with antiSMASH version 6.0 [7] extracted the complete set of gene clusters and genes involved in non-ribosomal and ribosomal synthesis of secondary metabolites previously identified in FZB42 Table 2. shows the potential to synthesize an impressive number of different secondary metabolites in *B. velezensis* strains BP1.2A, BT2.4, and FZB42.

**Table 1:** General genomic features of *B. velezensis* BP1.2A (CP085504.1), and BT2.4 (CP085505.1) compared with FZB42 (NC_009725.2). Methods used for generating the data are set in brackets (PGAP, RAST, EDGAR). Differences to FZB42 are labelled in red letters.

*Attributes*	BP1.2A	BT2.4	FZB42
Genome size (bp)	3,916,868	3,922,686	3,918,596
G+C%	46.5	46.5	46,5
Number of genes (PGAP)	3871	3870	3855
CDSs total (PGAP)	3753	3752	3734
CDS core genome (EDGAR)	3633	3633	3633
CDS pan genome (EDGAR)	3757	3757	3757
RNA genes (RAST)	118	118	118
rRNAs (PGAP)	27	27	29
tRNAs (PGAP)	86	86	88
ncRNAs (PGAP)	5	5	4
Pseudo genes (PGAP)	71	69	59
Number of coding sequences (RAST)	3939	3946	3938
Number of Subsystems (RAST)	324	324	324

*Subsystem Feature Counts*			

Cofactors, Vitamins, Prosthetic Groups, Pigments	147	147	147
Cell Wall and Capsule	73	73	73
Virulence, Disease and Defense	38	38	38
Potassium metabolism	3	3	3
Miscellaneous	24	24	24
Phages, Prophages, Transposable elements, Plasmids	0	0	0
Membrane Transport	42	42	42
Iron acquisition and metabolism	25	25	25
RNA metabolism	63	63	64
Nucleosides and Nucleotides	95	95	95
Protein Metabolism	209	209	211
Cell Division and Cell Cycle	6	6	6
Motility and Chemotaxis	42	42	42
Regulation and Cell signaling	28	28	28
Secondary Metabolism	6	6	6
DNA Metabolism	63	63	63
Fatty Acids, Lipids, and Isoprenoids	53	53	53
Nitrogen Metabolism	20	20	20
Dormancy and Sporulation	91	91	91
Respiration	40	40	40
Stress Response	43	43	43
Metabolism of Aromatic Compounds	12	12	13
Amino Acids and Derivatives	299	300	301
Sulfur Metabolism	6	6	6
Phosphorus Metabolism	12	12	12
Carbohydrates	215	215	215

**Table 2:** Detection of gene clusters involved in synthesis of secondary metabolites in the genomes of *B. velezensis* BP1.2A (CP085504), and *B.velezensis* BT2.4 (CP085505). For comparison FZB42 (CP000560.2) was also analyzed. Similarity to known metabolites listed in the MIBiG 2.0 repository [8] is indicated.

Region	CP085504		CP085505		CP000560.2		Similarity	
Surfactin	318,208	383,067	318,208	383,067	322,723	387,582	95%	BGC0000433
Plantazolicin	717,159	740,336	717,099	740,276	721,674	744,851	100%	BGC0000569
Ketoacyl:ACP synthase	935,682	976,926	935,298	976,542	940,739	981,983	100%	Bacillus
Squalene/phytoene synthase	1062,552	1079,781	1062,168	1079,397	1074,783	1075,523	100%	Bacillus
Macrolactin H	1366,841	1453,226	1366,457	1452,842	1371,897	1458,282	100%	BGC0000181
Bacillaene	1676,755	1777,357	1676,371	1776,973	1681,811	1782,413	100%	BGC0001089
Fengycin	1866,123	1903,373	1865,739	1902,989	1871,179	1908,429	100%	BGC0001095
Bacillomycin D	1907,878	1963,948	1918,319	1963,564	1923,759	1969,004	100%	BGC0001090
Squalene-hopene synthase	2010,880	2032,763	2010,496	2032,379	2024,219	2026,102	100%	Bacillus
T3PKS	2099,249	2140,349	2098,865	2139,965	2102,588	2143,688	100%	Bacillus
Difficidin	2269,142	2362,931	2268,758	2362,547	2344,012	2286,309	100%	BGC0000176
PK-5x Cys	2851,295	2900,808	2850,911	2906,712	2873,990	2884,225	88%	B.velezensis
Bacillibactin	3017,800	3024,927,	3023,696	3030,823	3021,021	3033,995	100%	BGC0000309
Amylocyclicin	3039,655	3045,228,	3045,551	3051,124	3043,470	3049,481	100%	BGC0000616
Bacilysin	3574,134	3615,552	3580,030	3621,448	3593,882	3599,780	100%	BGC0001184

**Fig. 1:**
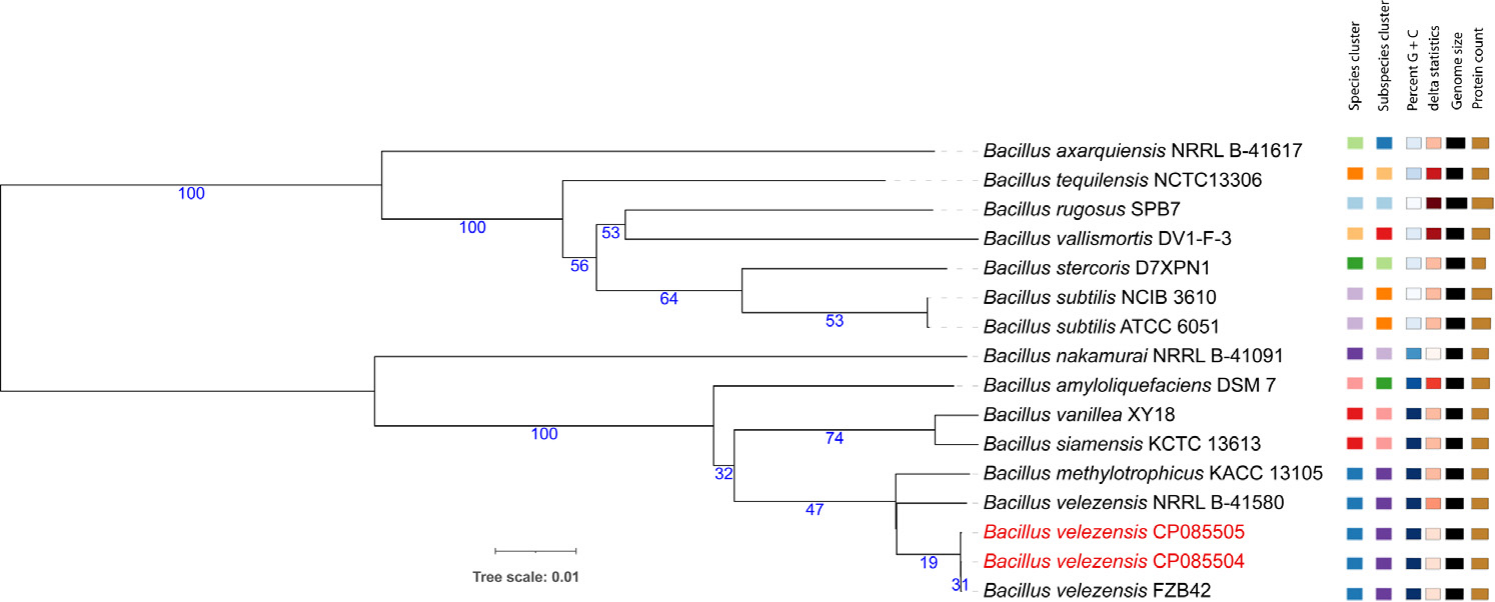
Phylogenetic tree of *B.velezensis* strains BP1.2A (CP085504), and BT2.4 (CP085505) labelled in red letters. The tree, based on whole genome sequences, was inferred with FastME 2.1.6.1 [9] from GBDP distances calculated from genome sequences. ^The branch lengths are scaled in terms of GBDP distance formula^*^d^5*^. The numbers below^ branches are GBDP pseudo-bootstrap support values from 100 replications, with an average branch support of 57.3%.

**Fig. 2:**
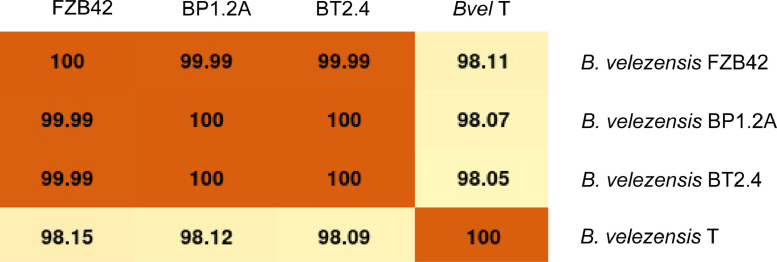
Pairwise comparison of the genomes of *B. velezensis* BP1.2A, and BT2.4 with *B. velezensis* FZB42, and the type strain of *B. velezensis* CCUG 50,740 using ANIb [11].

The phylogenomic analysis supported by TYGS [10] reveals that BP1.2A, and BT2.4 are representatives of the species *B. velezensis* (Fig. 1). Differences to *B. velezensis* FZB42 were not detected when the genomes were pairwise compared using ANIb [11] (Fig. 2) indicating their close relationship, despite that the sites of their isolation (Vietnam and Germany) are very remote from each other.

**Fig. 3:**
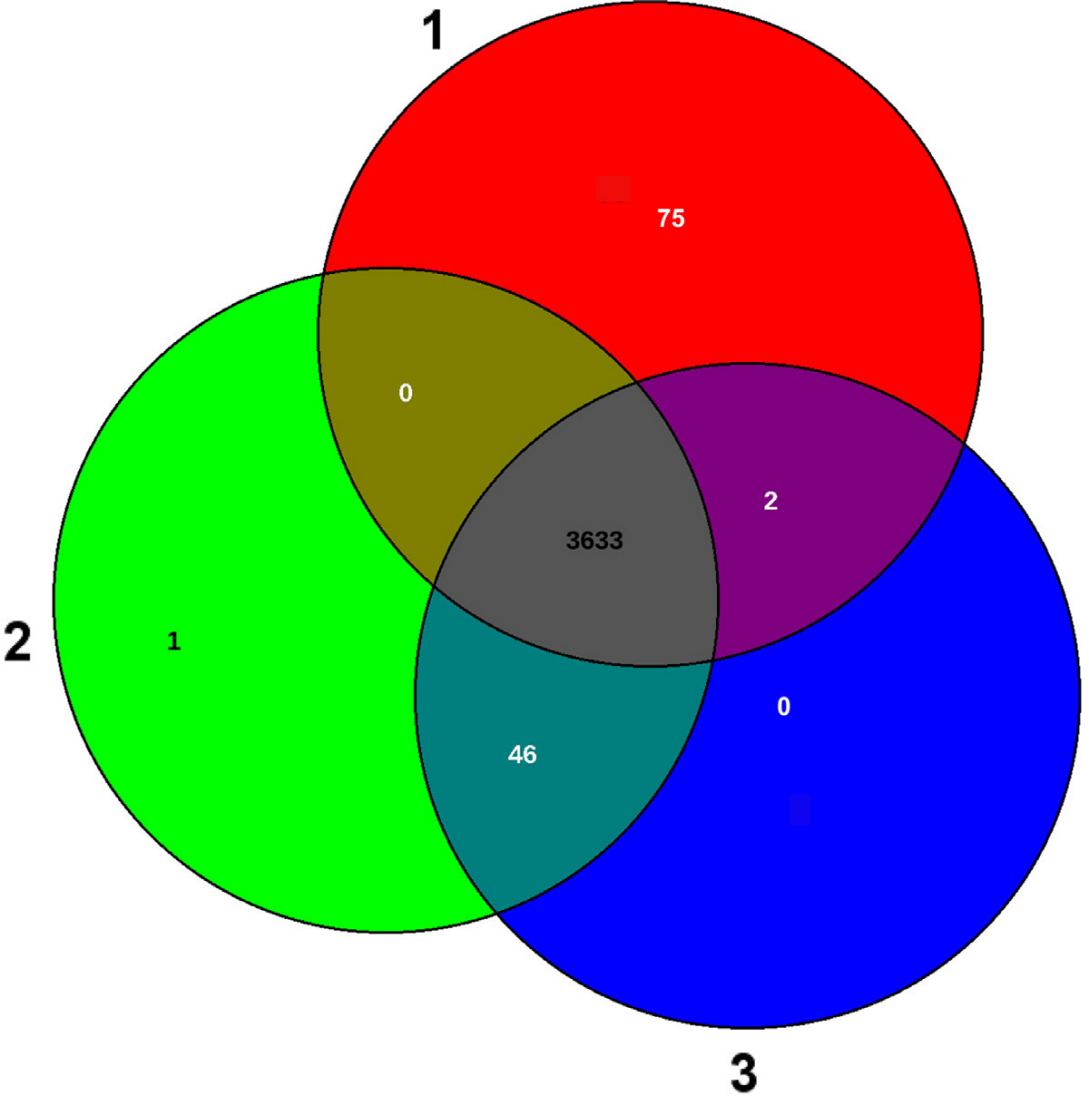
Venn diagram of the genomes of FZB42 (1), BP1.2A (2), and BT2.4 (3). Please note: The singleton numbers don´t necessarily correspond to the numbers in the “Singleton” interface (Table 3). The Venn diagram constructed with EDGAR shows the number of best hits between subsets of genomes. But: A gene without reciprocal best hit to another genome is not necessarily a singleton [12].

Table 3 and the Venn diagram presented in Fig. 3 summarize the comparison of the whole genome sequences of BP1.2A, and BT2.4 with FZB42. The three strains share a core genome of 3633 CDS. There is only one additional CDS (encoding a hypothetical protein) in BP1.2A, when compared with BT2.4 suggesting that both strains are identical or nearly identical clones, and the observed difference is due to sequencing error(s). Slight differences were detected, when the genomes were compared with FZB42. BP1.2A, and BT2.4 harbored 41 or 40 CDS, respectively, not occurring in the FZB42 genome. *Vice versa*, FZB42 harbored a total of 67 singletons, not present in the Vietnamese strains (Table 3). The slight differences to the numbers given in the Venn diagram (Fig. 3) are due to the different methods applied, as explained in the legend to Fig. 3.

**Table 3:** Sequence comparison of BP1.2A, and BT2.4 with FZB4242 using blastn, and ANIb [11]. The italic numbers set in brackets indicate the overlap of the sequences used in the comparison. Analysis of singletons was performed with the EDGAR software package [12].

*ANIb comparison*	BP1.2A (CP085504.1)	BT2.4 (CP085505.1)	FZB42 (CP000560.2)
BP1.2A	*	100 (99.74)	100.00 (99.64)
BT2.4	100.00 (99.67)	*	99.99 (99.58)
FZB42	100.00 (99.64)	99.99 (99.61)	*

*BLASTN comparison*	Query BP1.2A	Query BT2.4	Query FZB42

BP1.2A cover	100	99.854%	98.877%
BP1.2A identities	100	99.995%	99.989%
BP1.2A different nts	0	184/3,916,940	426/3,874,585
BP1.2A gaps	0	74/3,916,940	102/3,874,585

BT2.4 cover	100%	100%	99.866%
BT2.4 identities	99.996%	100	99.993%
BT2.4 different nts	174/3,916,868	0	274/3,911,604
BT2.4 gaps	25/3,916,868	0	21/3,911,604

FZB42 cover	99.697%	98.026%	100
FZB42 identities	99.987%	99.990%	100
FZB42 different nts	490/3,904,992	382/3,845,221	0
FZB42 gaps	182/3,904,992	192/3,845,221	0

*Singletons (CDS)*	BP1.2A	BT2.4	FZB42

BP1.2A	*	1	41
BT2.4	0	*	40
FZB42	67	67	*

## 2 Experimental Design, Materials and Methods

### 2.1 Strain growth conditions and DNA isolation

Cultivation of the Bacillus strains and DNA isolation have been previously described [1].

### 2.2 Genome sequencing, assembly, and annotation

Short-read sequencing was conducted in LGC Genomics (Berlin, Germany) using Illumina HiSeq in a paired 150 bp manner. Default parameters were used for all software unless otherwise specified. The short reads were trimmed and filtered using fastp [12] on default settings. Long-read sequencing was done in house with the Oxford Nanopore MinION with the flowcell R9.4.1 and prepared with the Ligation Sequencing Kit (SQK-LSK109). The samples were sequenced 48 h and basecalled afterwards by Guppy v3.1.5. Long reads were trimmed using Porechop (https://github.com/rrwick/Porechop, v0.2.4) and filtered using Filtlong (https://github.com/rrwick/Filtlong, v0.2.0) on default settings. De-novo assemblies were generated by using the hybrid-assembler Unicycler v0.4.8 [13]. The short-read assembly was done by SPades v3.13.0 [14] without read correction and normal bridging and the long-read assembly was done by racon v1.4.20 [15]. The quality of assemblies was assessed by determining the ratio of falsely trimmed proteins by using Ideel (https://github.com/phiweger/ideel).

### 2.3 Phylogenomics

The genome sequence data were uploaded to the Type (Strain) Genome Server (TYGS) for a whole genome-based analysis [10]. All pairwise comparisons were conducted using GBDP, and 100 distance replicates were calculated each. The resulting intergenomic distances were used to infer a balanced minimum evolution tree via FASTME 2.1.6.1 [9]. The tree was visualized with iTOL (https://itol.embl.de/#).

## Ethics Statements

This work did not contain human subjects, animals, cell lines or endangered species.

## CRediT authorship contribution statement

**Christian Blumenscheit:** Investigation, Methodology, Data curation, Software, Writing – original draft. **Jennifer Jähne:** Investigation, Methodology, Data curation. **Andy Schneider:** Investigation, Methodology. **Jochen Blom:** Software. **Thomas Schweder:** Conceptualization, Supervision. **Peter Lasch:** Conceptualization, Methodology, Supervision. **Rainer Borriss:** Conceptualization, Writing – review & editing.

## Declaration of Competing Interest

The authors declare that they have no known competing financial interests or personal relationships that could have appeared to influence the work reported in this paper.

## Data Availability

Bacillus velezensis strain BP1.2A chromosome, complete genome (Reference data) (NCBI GenBank).
